# Supplementary figures and images for: Light and Potassium Improve the Quality of Dendrobium officinale through Optimizing Transcriptomic and Metabolomic Alteration
Source: Molecules. 2022 Jul 29;27(15):4866. doi: 10.3390/molecules27154866 (PMC9369990; doi:10.3390/molecules27154866)

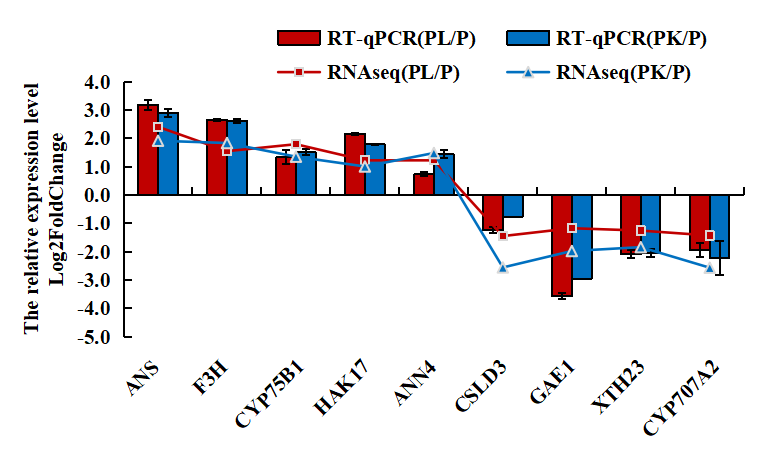

Supplement: Supplementary file 1 [file molecules-27-04866-s001.zip › Supplementary Files/Fig. S1 The expression pattern of 9 DEGs determined by RT-qPCR and RNA-seq.png]

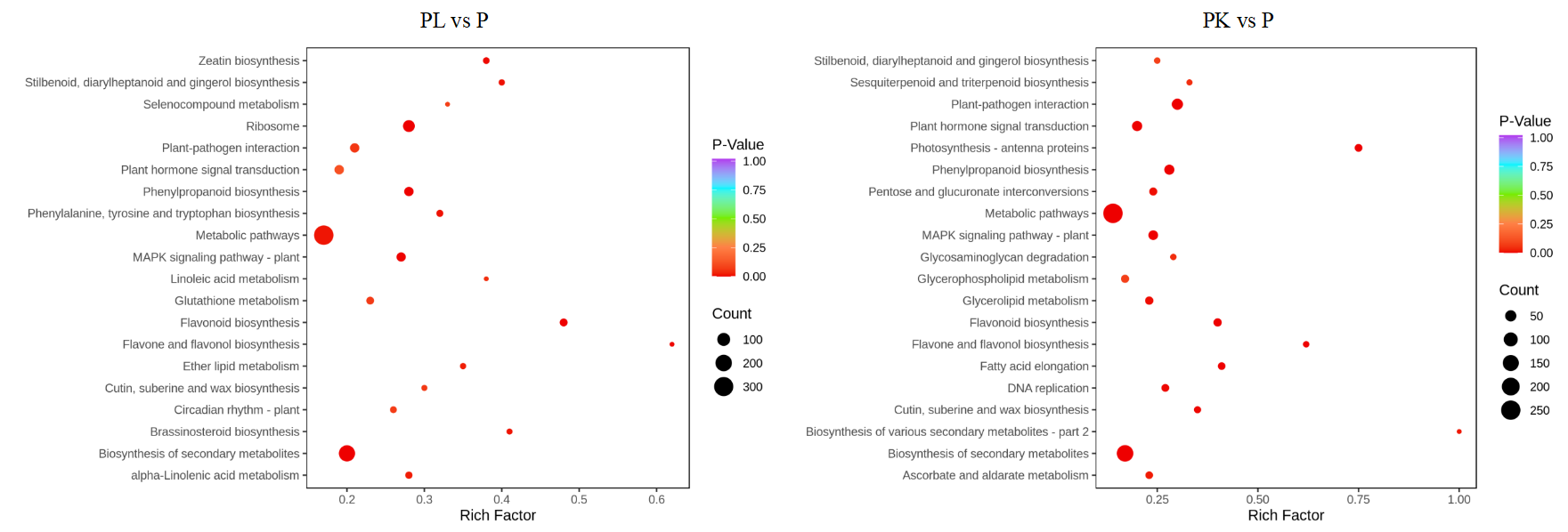

Supplement: Supplementary file 1 [file molecules-27-04866-s001.zip › Supplementary Files/Fig. S2 The top 20 KEGG-enriched pathways of DEGs in light- or K-treated D. officinale.png]

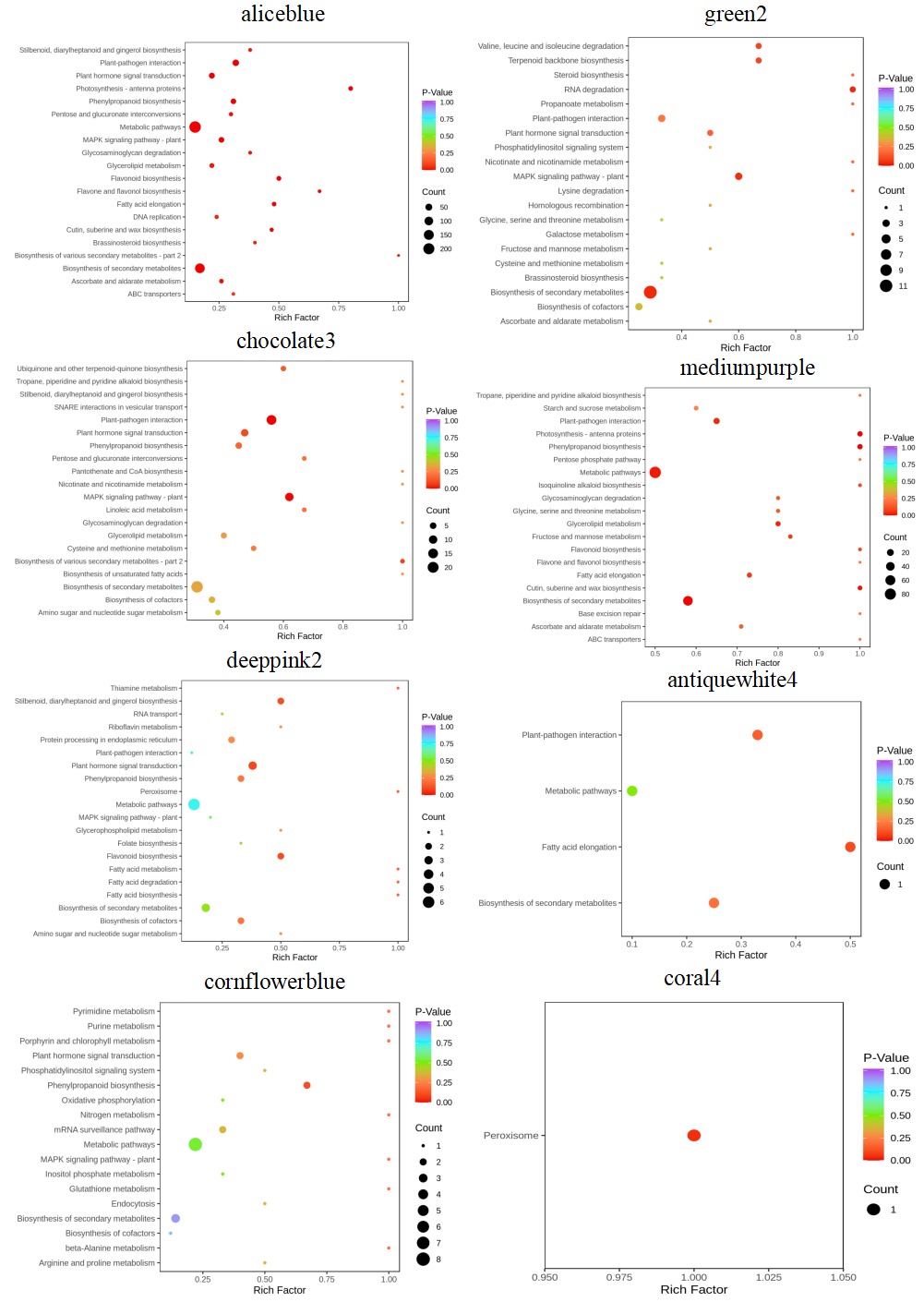

Supplement: Supplementary file 1 [file molecules-27-04866-s001.zip › Supplementary Files/Fig. S3 The top 20 KEGG-enriched pathways of DEGs in 8 coexpression modules.png]
